# Cincinnati Academy of Medicine

**Published:** 1880-07

**Authors:** 


### Reports of Societies.

### CINCINNATI ACADEMY OF MEDICINE.

*Rupture of the Perineum.*—Dr. Tate read a paper on "Rupture of the Perineum." The paper opened with a review of personal opinions on this subject in Great Britain and this country, drawn from conversation with medical men and the perusal of obstetrical journals, from which the conclusion was fairly drawn that there is still a great lack of information in regard to it.

The essayist then referred to the remarkable differences of opinion among physicians as to the causes of this disease, the means of preventing it, the effects which it left on the female, and as to the best methods of treatment.

The statement was then made that all our best English and American authorities represented that this rupture was of rare occurrence, even Leishman stating that anything beyond a rupture of the fourchette was seldom to be found.

The author then stated that this subject had been under examination in his department of the City Hospital for the last two years, during which time some two hundred women had been delivered at full time in his service. Of this number 142 were primiparæ and 59 multiparæ. The nurses and internes had been directed to examine every case ocularly after delivery, and if there were any rupture it was to be reported for treatment. This method of investigation revealed the fact that in two hundred cases there had been seventy-five cases of rupture of the perineum; seventy of which had been among the primiparæ, and five among the multiparæ. This is about equal to thirty-seven per cent. of the whole; fifty per cent. of primiparæ and eight per cent of multiparæ.

The paper then referred to the evils resulting to the mother, especially the pulling away of the buttress supporting the vaginal wall; and the fact that sterility would likely be produced by the incapacity of the vagina any longer to retain the seminal fluid in close proximity to the os uteri.

The essay next referred to the causes of rupture, and stated as the result of the observations of the author, the decided impression that by far the most frequent of these was rapid delivery and, above all, the large size of the child's head.

After reviewing the different methods employed to prevent rupture, such as support by the hand against perineum, notching, support by introducing the fingers in the rectum, etc., he came to the conclusion that inasmuch as the position of the head in relation to the pubis could not be essentially changed—that all means of support or assistance were useless, except those which would either prevent the too rapid descent or relax the vulva. The former he thought could be influenced by pressure directly with the hand against the head, and by withdrawing voluntary efforts to bear down; and for the latter, he thought reliance could be placed on chloroform. When a rupture had occurred, he was decidedly in favor of immediate treatment. With this view various methods had been employed, and for the most part, some plan of restoration by the aid of sutures was used.

For his part he preferred in all cases of rupture extending no further than the sphincter, the use of the serrefine. If the sphincter were also torn open, then, in order to draw the fibres of the muscle together and hold it, a stitch of silver wire was in addition to be employed.

The time and mode of operating was then explained, its advantages shown, and in order to insure success, especial emphasis was laid on the necessity of operating early, the use of an instrument without any cutting points, and of putting the arms well down on the sides of the lacerated walls.

As illustrating the subject and the results of treatment, he gave the record of his last term of service in the City Hospital. During this period 50 women had been delivered—35 primiparæ and 15 multiparæ. Among them there had been 18 ruptures; five extending to the sphincter, ten half-way across, and three to a less distance. Serrefines were employed in fourteen cases; in twelve, a complete union; in one, a partial success; in one, a complete failure. This last was the case of a woman who had b en in labor a long time with the head resting on the perineum. After delivery a slough formed along th the edge of the wound, and, of course no union could take place.

Dr. Whittaker said that the experience of the essayist as to the frequency of perineal rupture coincided with his own; and he could only explain that of the old physicians who considered it rare by their neglect of observation. One will never know the truth about it, if he relies on the statement of the nurse, still less if he relies on the patient herself. The nurse always says it is all right. A physician who does not himself inspect the condition of the perineum knows nothing about it, and his opinion as to the frequency of the accident is worth nothing.

It was not the head in the vast majority of cases that ruptured the perineum. The speaker had watched the perineum closely, in forceps deliveries, an observation he thought absolutely imperative to make always, had kept it dried away with a towel during the process of the slowest possible extraction and had never yet seen it rupture under distension. What bursts the perineum is the shoulders. The speaker was so convinced of the fact that he often delivers the sacral shoulder first, by sweeping the sacral arm over the chest, before allowing the whole body to be born. He has seen cases where after the greatest precaution with the head and shoulders a perineum so far safe, had been torn back to the sphincter by the hips.

Any one who practices much among women would have impressed upon him the necessity of saving the perineum. The speaker did not believe the evils of perineal rupture to have been exaggerated in the least. He could scarcely recall a case of malposition of the uterus or of catarrhal inflammation in a patient whose perineum had been preserved intact. He would only emphasize the point that rupture of the perineum breaks away the dam, which retains the semen about the os and cervix uteri. Every gynecologist knows that after penetration through the sphincter, the finger enters a wide soft sac, which is the cavity, or bay, as Kristeller remarks, in which the seminal fluid lies. Laceration of the perineum removes the natural bar to the escape of this fluid and thus not infrequently causes sterilty. So if the modern gynecologist would transfer his field of operations from the too much abused uterus to the outside perineum, he would oftener succeed in the relief of various uterine maladies, many of which are occasioned by the neglect of the obstetrician at the time of the labor.

As to any preventive of such laceration by the application of hot water, or by forcibly pushing back the child, the speaker was wholly incredulous. Hot water will not soften a rigid perineum to any appreciable degree. The distension which the skin experiences under the longest continued poultice is wholly insignificant. It is yet a question among physiologists whether the skin absorbs any water at all after a prolonged bath. Pushing back the head would not be tolerated by the patient. It would be looked upon as a brutality and the attempt resented. Moreover, if attempted it would be entirely inefficacious. The obstetrician might push with such force as to push back the body of the mother and it could not arrest the progress of the labor. Nor did the speaker have much faith upon the relaxation of chloroform, for the detension is not active, but passive. And though chloroform might relax the *vis a tergo* force, it could not relax the skin. Most patients get chloroform now and it does not save the perineum.

Rupture of the perineum does not occur so often among the lower classes. A gross construction of the parts saves them. The lower animals scarcely suffer from it at all.

Dr. Palmer remarked that those who contended that rupture of the perineum was uncommon, never made any special examination of the parts to determine the matter. This was the only explanation to be offered for the statements of practitioners with large experience holding such views.

It was probably no exaggeration to say that rupture of the perineum to the first degree occurred in primiparæ in nearly ninety per cent. of deliveries, and to the second degree in thirty per cent. In his own experience ruptures occurred in spite of efforts timely directed to prevent. Some ruptures in certain cases must inevitably follow by virtue of the size of the vulvar orifice, its direction, the swollen and œdematous condition of the parts, the rigidities, the unfavorable position of the head, and its great size.

Careful examination of the vagina and perineum should be made by inspection as well as by touch, after every delivery to see the exact state of the soft parts. No lady will object to this examination, properly made, its object having been previously stated. The position on the side is best adapted for this.

The so called support of the perineum is a misnomer. The sole object of attention to the perineum is to retard the movement of the head or protruding part when precipitous delivery is threatened, and above all, by judicious application of the hand on the posterior surfaces of the perineum, or with fingers in the rectum after the manner of Goodell, to direct the presenting part as far as possible forward under the pubic arch. This is meant by support of the perineum. It would be better if the term were abandoned.

As to the part of the fœtus which produced the rupture, it was his experience that while either head and shoulders might do the mischief, in a considerable proportion of instances, the commencement of the laceration was made by the head and continued by the shoulders. It was an easy matter for the rupture to be extended after a solution of continuity was commenced. Thus, the greater portion of the laceration was produced in this way, and partly because less care was usually devoted to carrying the shoulders and body of the child forward away from the perineum.

These ruptures usually commence just within the fourchette, along the posterior vaginal wall, and extend in either direction, sometimes very extensively up the vagina.

The use of the serrefines, with which the essayist had such good results, he believed was very good for ruptures of the perineum in the first degree, but where the lacerations were more extended, and more particularly when along the vaginal wall, these instruments were inadequate to accomplish complete coaptation along the whole rent. They would doubtless secure complete union where fastened, that is, along integumentary portions of the rent, but left the upper surfaces still gaping, free to absorb the putrid material, heal imperfectly by granulation only, and did not restore the triangular perineal body. Sutures either of silk or silver wire, well placed, alone could restore a properly shaped perineum. As to the propriety of the primary operation there could be no doubt. The weight of authority was in favor of it; common sense favored it. Its importance in well marked ruptures was attested by a study of the anatomy of the parts, the functions they had had to perform, and finally by clinical results seen by a want of attention to it. Easy of execution, so satisfactory in results, the primary operation was simply a matter of duty.

Dr. Julia Carpenter said that she had spent a month in the obstetric department of the General Hospital at Vienna, where the average number of confinements during that month was about eighteen per day. During her stay she had seen but one case of rupture of the perineum. This was an instrumental case, and for its relief sutures were applied after several hours.

Whenever a rupture was feared one or more lateral incisions were made, and by this means the laceration was avoided.

Dr. Reamy could fully agree with the author of the essay as to the frequency of perineal lacerations. He was convinced that the statistics of Schrœder, placing the accident at 34.5 per cent. of deliveries, are below rather than above the mark. He considered it safe to affirm that 40 per cent. of all first deliveries are attended by the accident in one or another degree. More freqnently only in the first degree. The accident to the third degree opening in the bowels is, in the hands of a competent practitioner, very rare. The declaration of physicians of large experience that they had never encountered a case of laceration of the perineum, could be easily explained. These gentlemen had simply never encountered a case in the third degree, and had overlooked the milder cases occurring in their hands. He had himself supposed that no case of lacerated perineum had occurred in his practice up to the time when he had delivered one thousand women. No case to the severe degree had occurred, and he, as others, had not appreciated the simple cases. He was convinced of the fact that women in the country districts, whose modes of life are quite different from those in the cities, are not nearly so liable to the accident.

The speaker could not reconcile the statements made by Dr. Carpenter as to the large number of primiparæ delivered without rupture in the Vienna Hospital during her stay there. The reports of that hospital had always shown a lower per cent. of lacerations than of any other hospital in the land, but all critics agreed that these reports had been too favorable, probably because the observations were largely made by midwives, who, if fully competent, would still be under the influence of selfish motives that would depreciate the value of their statistics. But Dr. Carpenter's reports were still more favorable. He could only assure Dr. C. that eight or ten years' further experience in private practice would not show such gratifying results. At least if so she would be more fortunate than her neighbors in the management of cases.

The speaker considered laceration to the third degree due to neglect or incompetency on the part of the attendant, but to the first degree often and to the second degree occasionally unavoidable in the most skilled hands. He would not enumerate the generally conceded causes of laceration. As to the agency of the head and shoulders, he had seen cases where the damage was done by the head alone, the shoulders being delivered with ease through the now enlarged outlet without further injury to the parts. In other cases, and perhaps more frequently, the shoulders had done the mischief. Mathews Duncan had pointed out how frequently it is that the damage commences at the vaginal orifice—a point, roughly speaking, about the size of the hymen, and not at the posterior commissure.

He could not assent to the proposition that nothing could be done by the accoucheur to prevent rupture. No one could be found now probably who would advocate direct pressure, so called support by the hand upon the perineum. This is of course injurious practice. Introducing two fingers into the rectum, and thereby at the same time relaxing the perineum, and carrying the head closely forward under the pubic arch has the high authority of Goodell for its support. The speaker felt great reluctance in opposing a practice endorsed by one so distinguished as Dr. Goodell. Nevertheless, having tried it, he was not pleased with it. It was disgusting to the patient, caused pain and severe reflex irritability, adding to the woman's suffering. Moreover, if the pressure is not very gently made upon the rectal wall, stretched as it is already, damage may result in the tissues. Again the manipulation did not, in the opinion of the speaker, relax that portion of the perineum which is threatened nearly so well as it may be done by another method, viz., applying the hand back of the anal opening and carrying all the parts forward together. The external anal sphincter, as all know, arises from the coccyx, and crossing in the perineal body, passes on either side of the vulva opening. Now the coccyx being movable and being at the time thrust backwards could, by the manipulation referred to, be carried forward so as to relax the perineum. The fact that the *syhincter vaginæ* muscles arise in the perineal body made this more certain. Moveover, this movement shortens the stretch of the traverse perineal muscle. The speaker had on several occasions watched with the eye the perineum when stretched to the point of danger, and the lessening of the tension under the manipulation indicated could be unmistakably appreciated.

The forceps have been accused, and perhaps in some instances justly, of causing lacerations. In his experience by no physical means could the too rapid advance of the head be prevented so well as by the forceps. With the instrument properly applied the operator has complete control of the head, and when it is upon the perineum, he can advance or retard it at will by the forceps. Moreover the head could be made to hug the pubic arch more effectually by this than by any other method. He would not, of course, advise the application of the forceps solely for the purpose of protecting the perineum, though he would consider such a course, in some cases, justifiable; but as it so happens that in many cases where the perineum is threatened the forceps are applied on other accounts, this method of protection was of great value.

But the remedy above all and over all against perineal laceration was *anæthesia.* Chloroform relaxes the perineum modifies precipitous pains and increases vaginal secretion.

The speaker could not agree with the essayist as to the applicability of serrefines to any cases but those of first degree. Here they were no doubt invaluable, but in all cases they could only secure imperfect repair, union would occur along the line of the skin, but this alone could do nothing toward avoidance of the serious damage that must ultimately follow imperfect repair of the whole perineal body.

He preferred in all cases the silver wire suture. He had formerly invariably ordered the bowels locked by opiates for a week. He now believed that in these cases it is quite as well to have them moved, at least by the third day, as by that time union would be accomplished. And this practice would avoid the danger of damage to the new tissue which might attend the first evacuation after the bowels had become filled with solid fecal matter. He would of course advise the immediate operation in nearly all cases of laceration.

Dr. C. O. Wright had about concluded that his experience was much the same as that of some country practitioners quoted who had never seen a case of laceration of the perineum. Since the explanation, however, of the classification of these accidents had been made, he believed he had seen some slight cases.

But the point he wished more particularly to bring out was the great disparity in the number of cases occurring under the observation of the essayest and under his own observation when a resident physician in the same institution. Ninety cases of confinement had occured to the speaker's knowledge without a single case of perineal rupture. In his practice he had never failed to support the perineum, and he believed that his succes in having no cases of rupture of the second degree was due principally to that fact.

Dr. Conner had never seen any but slight ruptures. He was satisfied that it was the shoulders and not the head that lacerates the part. Serrefines would not answer the indications for treatment except in slight cases. He asked why should the catheter be used in these cases and the bowels be kept constipated. In a fair proportion of operations for ruptured perineum, women could pass water without the catheter. He thought its use unnecessary in lithotomy and much less so in the operation in question. In cases demanding the employment of the catheter some form of soft catheter might be introduced and retained.

The bowels, he thought, should not be locked up for a week or more, but left to take care of themselves. There was often danger of tearing open the parts by the passage of hardened feces; and, in fact, constipation was more to be guarded against than diarrhœa.

Dr. Vance wanted to draw the attention of the Academy to a point based on his own personal experience. In his first case of obstetrics, while in the Bellevue Hospital, he had had a rupture which extended about two inches up in the bowel, occurring with a child weighing only eight pounds. This was the only case of complete rupture he had seen in a large number. He mentioned eighteen cases of primiparæ in German women which occured with a single case of rupture, and he thought that nationality and the state of health of the individual had something to do with the frequency of the accident. Notwithstandihg the experience related above, he thought the average of cases of rupture at the Bellevue was as high as elsewhere.

As to the immediate cause of the laceration, he thought it was commenced with the head and continued by the shoulders.

He had had very extensive experience as an expert witness in suits for malpractice, and in all his observation he had but one case to recall where such a suit had been instituted for laceration of the perineum. In this respect obstetricians were more fortunate than surgeons.

Dr. Ransohoff stated in explanation of the rarity of laceration of the perineum at the Vienna General Hospital, that there were more multiparæ than primiparæ to be found in the wards. Large numbers of married women were in the habit of going there to be confined, and he considered this as probably going far to explain the anomalous statistics furnished by that institution.

Dr. Tate after thanking the members for the interest they had taken in his paper, said there were two or three objections made to the views he had expressed, which justice to the paper would seem to require that he should answer.

It had been said that no pressure made by the hand against the head could have any influence in arresting its descent. Now he thought it would be the general consent of obstetricians that nature, in general, furnished the uterus with just about enough power to enable it to expel the fœtus—there was very little extra endowment here—and theref re if the hand of the *accoucher* were planted in opposition to the descent of the head, it seemed to him it would most naturally delay its exit. He could not see either why it might not be just as effectual, and assuredly more appropriate, in such cases than the forceps. This instrument certainly has the credit more of producing than preventing ruptures. Some gentlemen had expressed the opinion that the shoulders, more frequently than the head, is the cause of rupture. He did not see how any one could maintain this position, unless he was willing to affirm as a mathematical axiom, that when a larger body and a more unyielding one has passed through an orifice, and is succeeded by a smaller, a softer, and more yielding body, the latter will be the one which will tear through the opening. He thought, however, that it was not an unusual thing, after the head in passing had started a rupture, for the shoulders in coming through to extend this rupture still further backwards.

The paper had offered some urgent reasons why *serre-fines* should be employed in treating recent ruptures. In objection to them it had been said that although they may unite the skin and superficial tissues, they fail to bring together the sides of the body of the perineum. It will be noticed, however, that the paper with special reference to this particular, directs that an instrument should be chosen which should have sufficient strength of grasp, and with long arms, so that they can be pushed well down on the sides of the body of the perineum. Now when this was done, he felt sure that the torn sides of the body must be brought in direct apposition, and held there, and that too in a far more natural relation than could be produced by any stitch, for this must always produce a pucker.

In regard to the constipation induced by opium in these cases, it seemed to him that the point was well taken; for there certainly had, in some cases, been subsequent tearing away of the union by the passage of hardened feces. He would make a suggestion as to whether this difficulty might not be obviated when the time arrived to open the bowels, by injecting warm water and animal bile to soften the feces.

*High Temperature in Scarlet Fever.*—Dr. Cleveland reported a case of convulsions in a child, attended with remarkably high temperature. When first seen the child had a temperature of 105° F., and suspecting indigestible matters in the stomach to be the cause of its sickness, the speaker ordered an emetic. At his second visit the spasmodic twitchings of the muscles were still present, and the thermometor showed 110° F.

The speaker was of the opinion that the temperature was still higher, as the top ol the scale was reached and the instrument would record no more. The case was supposed to be one of scarlet fever.

The child died.

The speaker observed that the highest temperature from which he had seen a case recover was 107° F., in a child with scarlet fever.—*Cincinnati Lancet and Clinic.*

